# TITAN: inference of copy number architectures in clonal cell populations from tumor whole-genome sequence data

**DOI:** 10.1101/gr.180281.114

**Published:** 2014-11

**Authors:** Gavin Ha, Andrew Roth, Jaswinder Khattra, Julie Ho, Damian Yap, Leah M. Prentice, Nataliya Melnyk, Andrew McPherson, Ali Bashashati, Emma Laks, Justina Biele, Jiarui Ding, Alan Le, Jamie Rosner, Karey Shumansky, Marco A. Marra, C. Blake Gilks, David G. Huntsman, Jessica N. McAlpine, Samuel Aparicio, Sohrab P. Shah

**Affiliations:** 1 Department of Molecular Oncology, British Columbia Cancer Agency, Vancouver, BC V5Z 1L3, Canada;; 2 Bioinformatics Training Program, University of British Columbia, Vancouver, BC V5Z 4S6, Canada;; 3 Centre for Translational and Applied Genomics, Vancouver, BC V5Z 4E6, Canada;; 4 Department of Computer Science, University of British Columbia, Vancouver, BC V6T 1Z4, Canada;; 5 Genome Sciences Centre, British Columbia Cancer Agency, Vancouver, BC V5Z 1L3, Canada;; 6 Genetic Pathology Evaluation Centre, Vancouver General Hospital, Vancouver, BC V6H 3Z6, Canada;; 7 Department of Pathology and Laboratory Medicine, University of British Columbia, Vancouver, BC V6T 2B5, Canada;; 8 Department of Gynecology and Obstetrics, University of British Columbia, Vancouver, BC V5Z 1M9, Canada

## Abstract

The evolution of cancer genomes within a single tumor creates mixed cell populations with divergent somatic mutational landscapes. Inference of tumor subpopulations has been disproportionately focused on the assessment of somatic point mutations, whereas computational methods targeting evolutionary dynamics of copy number alterations (CNA) and loss of heterozygosity (LOH) in whole-genome sequencing data remain underdeveloped. We present a novel probabilistic model, TITAN, to infer CNA and LOH events while accounting for mixtures of cell populations, thereby estimating the proportion of cells harboring each event. We evaluate TITAN on idealized mixtures, simulating clonal populations from whole-genome sequences taken from genomically heterogeneous ovarian tumor sites collected from the same patient. In addition, we show in 23 whole genomes of breast tumors that the inference of CNA and LOH using TITAN critically informs population structure and the nature of the evolving cancer genome. Finally, we experimentally validated subclonal predictions using fluorescence in situ hybridization (FISH) and single-cell sequencing from an ovarian cancer patient sample, thereby recapitulating the key modeling assumptions of TITAN.

Tumor progression follows the principles of clonal evolution (Nowell 1976). Accumulation of genomic alterations is patterned by phylogenetic branching, creating a substrate for natural selection. Invariably, this leads to the emergence of distinct cell populations (clones) with divergent genotypes and associated phenotypes (Aparicio and Caldas 2013). Here, we define a clone as a population of cells related by descent from a unitary origin and uniquely identified by the complement of fixed genetic marks comprising its clonal genotype. Genetic marks can consist of somatic mutations such as point mutations, genome rearrangements, copy number alterations (CNA), and loss of heterozygosity (LOH), of which CNA and LOH are the focus of this study. We define the cellular prevalence of a somatic mutation as the proportion of cells harboring an aberration in the overall (bulk) tumor cell population (Aparicio and Caldas 2013). Cellular prevalence can be measured approximately through sequencing a bulk sample, or more precisely in independent analysis of single cells (Navin et al. 2011). The dynamics of cellular prevalence of a mutation are reflective of growth (dis)advantages in the presence of treatment or microenvironment-induced selective pressures and are thus a useful indicator of the biology underpinning tumor progression.

The clonal evolution theory implies that extant clones are related genetically through a phylogenetic tree. In such population structures, cellular prevalence of a genetic alteration is generally a function of its evolutionary timing: High-prevalence mutations are acquired earlier than low-prevalence mutations. Thus, ancestral mutations are found at the root of the tree, whereas descendent mutations are situated toward the leaves. We explored the resulting patterns of alterations acquired after expansion of the ancestral clone, which generates three types of cells in a tumor sample: normal (nonmalignant) cells, tumor cells harboring the alteration, and tumor cells without the alteration. This concept applies to all forms of genomic aberrations, including CNA and LOH, despite a disproportionate emphasis on point mutations in the literature (Shah et al. 2009, 2012; Ding et al. 2012; Gerstung et al. 2012; Cibulskis et al. 2013; Landau et al. 2013; Larson and Fridley 2013; Roth et al. 2014). Indeed, tumors with diverse intratumoral patterns of CNA and LOH have been described in breast (Navin et al. 2011; Nik-Zainal et al. 2012), ovarian (Bashashati et al. 2013), renal (Gerlinger et al. 2012, 2014), and brain tumors (Sottoriva et al. 2013).

Whole-exome (WES) and -genome (WGS) sequencing of a single biopsy are emerging as the dominant experimental designs in large cohort studies of tumor genomic landscapes, with consortia such as the International Cancer Genome Consortium (ICGC) poised to generate on the order of 10,000 tumor-normal WGS libraries in the next few years (The International Cancer Genome Consortium 2010). Characterization of clonal populations from such data sets has been primarily focused on point mutations, which require targeted deep sequencing. Measuring cellular prevalences of CNA and LOH presents unique challenges because these events can span megabases, rendering targeted deep sequencing of alleles infeasible. Moreover, heterogeneous mixtures of cells in tumor biopsies present a major limitation in accurate interpretation of WGS data. CNA and LOH events present in only minor cell populations will have diminished statistical signals and thus are susceptible to false negative detection. Figure 1 depicts the observed read depth (top track) and allelic ratios (middle track) from subclonal deletions (*Deletion I* and *III*) and a high-prevalence clonal deletion (*Deletion II*), illustrating the distinct statistical signals arising from differences in cellular prevalence (bottom track). The degree to which CNA and LOH contribute to the inference of evolutionary dynamics cannot be estimated using the most current standard approaches. Methods for robust computational models of statistical signals emitted from multiple cell populations within a single tumor sample are therefore underdeveloped and represent a deficiency in the cancer genomics literature.

**Figure 1.**
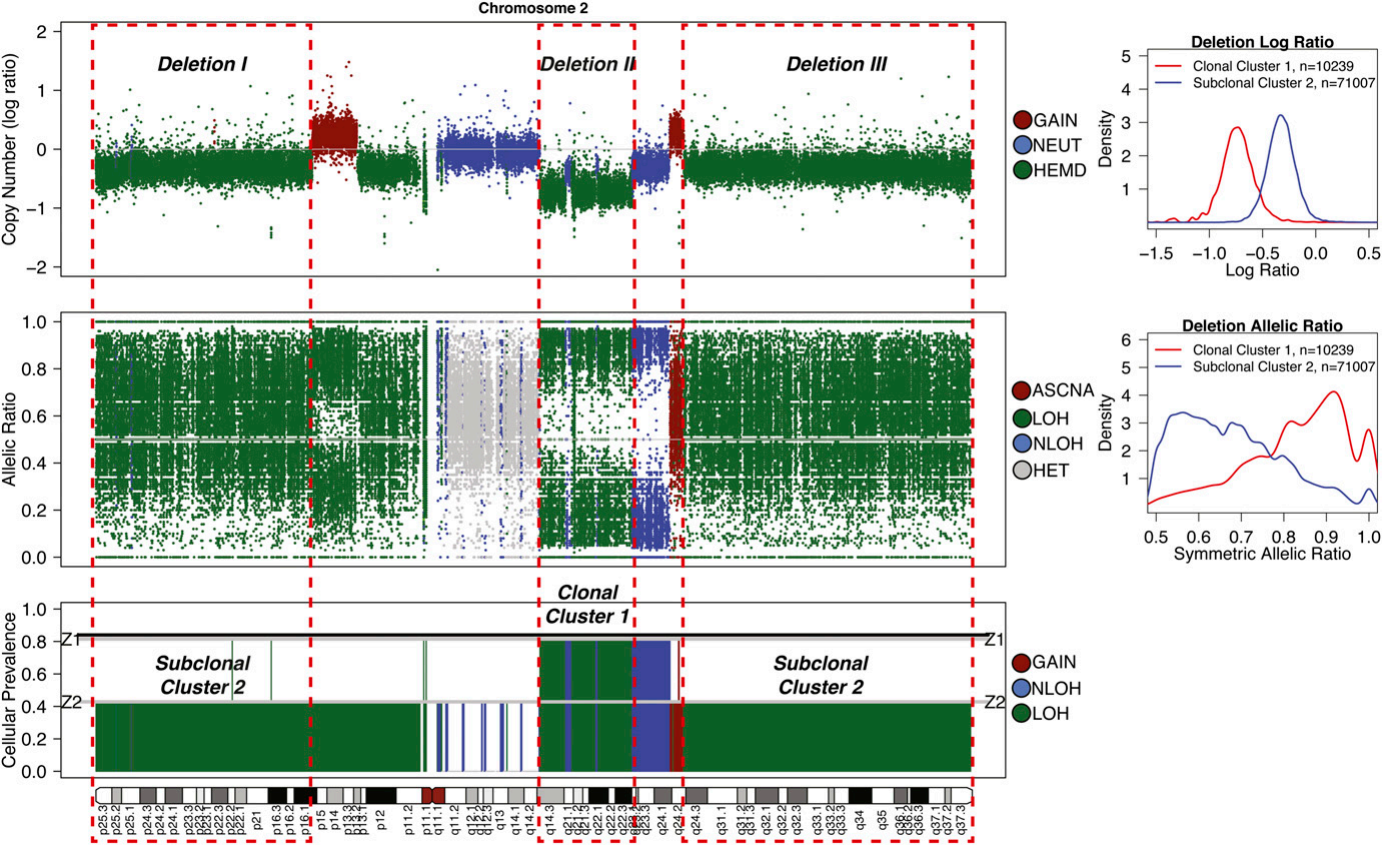
Detection of subclonal deletions in whole-genome sequencing data of a triple negative breast cancer genome. Copy number is represented as the log ratio of tumor and normal read depth. Discrete copy number status shown is predicted as a hemizygous deletion (HEMD; green), copy neutral (NEUT; blue), or gain/amplification (AMP; red). Allelic ratios are computed as the proportion of reads matching the reference genome. The LOH status shown is heterozygous (HET; gray), LOH (green), copy neutral LOH (NLOH; blue), or allele-specific gain/amplification (ASCNA; red). Subclonal deletions are observed to have a weaker log ratio signal that is closer to zero and shows less spreading in allelic ratios (Deletion I) compared to clonal deletions (Deletion II); the sample cellular prevalence estimates (proportion of sample) for “Deletion I” indicate it is in a subclonal cluster “Z2.” “Deletion I” and “Deletion III” are clustered into the same subclonal cluster because they share similar signals, and therefore the same cellular prevalence in the data. “Deletion II” is present in all tumor cells, indicated by being in the clonal cluster “Z1.” Tumor cellularity of 84% (normal contamination of 16%) is denoted with a black horizontal line. The average tumor ploidy (haploid coverage factor) was estimated as 1.66 by genome-wide analysis (*right*). The log ratio and symmetric allelic ratio (*max*(*reference reads*, *variant reads*)/*depth*) for Gaussian kernel densities are shown for all deletions on Chr 2.

We developed a novel probabilistic model called TITAN. The model simultaneously infers CNA and LOH segments from read depth and digital allele ratios at germline heterozygous SNP loci across the genome from tumor WGS data. For each alteration, we assume the event is segregated into the underlying population of three different cell types: normal cells, tumor cells containing the event, and tumor cells without the event (Fig. 2A). We estimate the cellular prevalence of the CNA/LOH with the assumption that co-occurring events will be represented in the same clones, resulting from “punctuated” clonal expansions (Navin et al. 2011; Greaves and Maley 2012). This motivates a clustering paradigm for statistical inference, allowing for increased power to detect weaker signals in the data across multiple loci and to distinguish sets of events at different cellular prevalences (Fig. 1). We integrated this approach in a generative, factorial hidden Markov model (HMM) framework. The approach borrows statistical strength across adjacent genomic loci induced by segmental CNA and LOH events spanning multiple contiguous SNPs (Methods).

**Figure 2.**
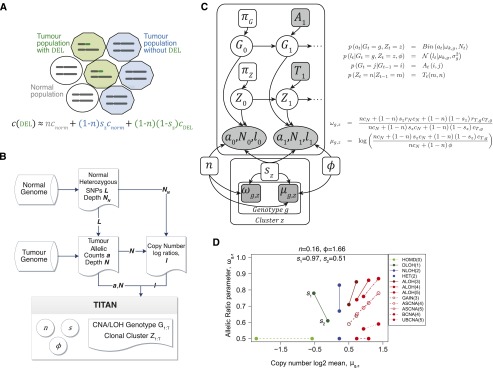
Description of the TITAN probabilistic framework. (*A*) Representation of the aggregate copy number signal from mixed populations in a heterogeneous tumor sample. *c* is the aggregate signal that is composed of three components: normal population (white circles), tumor populations with the deletion (green decagons) and without the event (blue decagons). *n* is the normal proportion; *s*_*z*_ is the tumor proportion for the *z*_*th*_ clonal cluster that *does not* contain the event; *c*_*norm*_ and *c*_*DEL*_ are normal and tumor copy numbers. Therefore, (1 − *s*_*z*_) corresponds to the proportion of tumor harboring the event, also defined as the tumor cellular prevalence of the *z*^*th*^ clonal cluster. (*B*) Analysis workflow for TITAN. Three inputs are required: (1) Heterozygous positions identified in the normal DNA predicted by genotyping tools such as SAMtools mpileup (Li et al. 2009); (2) reference counts *a* and read depth *N* are extracted at these positions from aligned reads in the tumor DNA sequence data; and (3) the tumor and normal read depths, *N* and *N*_*N*_, are normalized independently to correct GC content and mappability biases; log ratios *l* = log(*N*/*N*_*N*_) of the corrected read counts are computed. The output is the optimal sequence of CNA/LOH genotypes and clonal cluster memberships at each position. Model parameters for normal contamination *n*, tumor cellular prevalence *s*_*z*_, and tumor ploidy *φ* are estimated. (*C*) Probabilistic graphical model of TITAN. Shaded nodes are known or observed quantities; open nodes are random variables of unknown quantities. Arrows represent conditional dependence between random variables. Full details and definitions are in Methods and Supplemental Table 13. (*D*) Parameter trace of *ω*_*g*,*z*_ and *μ*_*g*,*z*_ when cellular prevalence varies. *s*_1_ and *s*_2_ are shown as the tumor cellular prevalence (i.e., transformed using 1 − *s*_*z*_). *n* is normal proportion and *φ* is average tumor ploidy. Each CNA/LOH genotype is shown (Supplemental Table 14) with the associated integer copy number in parentheses.

Our approach is distinct from related methods in the literature. Methods such as APOLLOH (Ha et al. 2012) and Control-FREEC (Boeva et al. 2012) model normal contamination from WGS of tumors, but do not jointly infer CNA and LOH in a unified statistical approach, nor do they explicitly account for multiple tumor subpopulations. SNP genotyping array-based methods, such as OncoSNP (Yau et al. 2010), analyze CNA while accounting for intratumoral heterogeneity in cancer samples but cannot be directly applied to WGS data. Recently developed approaches, ABSOLUTE (Carter et al. 2012) and THetA (Oesper et al. 2013), were designed with the aim of predicting subclonal CNA events specifically for tumor sequencing data. However, neither tool uses a complete model that provides segmentation analysis. Moreover, THetA analyzes subclonal CNA in the absence of allelic ratios, which results in the omission of LOH and allelic imbalance. Finally, OncoSNP-seq (Yau 2013) accounts for mixed populations in WGS data but does not model distinct clonal populations in a clustering approach, which is characteristic of punctuated expansions.

We present a rigorous evaluation of TITAN including: (1) single-cell sequencing and fluorescence in situ hybridization (FISH) experimental validation of predictions on WGS data from a high-grade serous ovarian tumor; (2) systematically engineered in silico mixtures with WGS data from multiple intrapatient samples; (3) artificially embedded CNA and LOH events in diploid chromosomes; and (4) 23 triple negative breast cancer (TNBC) genomes. We compared TITAN with four published methods to demonstrate, with quantitative benchmarking on the ground truth data sets, that TITAN has higher sensitivity to detect low cellular prevalence events without decreasing specificity and has the capacity to accurately estimate cellular prevalences. Application of TITAN to 23 TNBC samples shows that a substantial proportion of clonal diversity is captured in the CNA/LOH dimensions, with low cellular prevalence LOH impacting allele-specific expression inferred from matched RNA-seq data. Finally, results from FISH and single-cell sequencing validation experiments confirm that predicted CNA/LOH events were consistent with our key modeling assumption: Distinct cell populations can be identified through inference of CNA/LOH from WGS data. Together, these data show that the cellular prevalence profile of the copy number architecture from WGS provides an effective route to inferring clonal populations in patient tumor samples. Finally, we suggest how the deployment of TITAN in large-scale clinical studies can dissect the interplay between clonal evolution, DNA repair deficiencies, and response to therapy.

## Results

TITAN is a statistical model for predicting segmental CNA and LOH from matched tumor and normal WGS data. The input to the model is the full set of germline heterozygous SNP loci (identified from the normal sample) and the corresponding read depth and allele ratios at these SNP positions from the tumor. The output is a set of segmental CNA and LOH events, clonal cluster memberships, and estimated cellular prevalences (Fig. 2B). The *tumor* and *sample* cellular prevalences are defined as the proportion of the tumor cells and the proportion of the sample (including normal cells) that harbor a CNA/LOH event, respectively. The model is predicated on four main assumptions: (1) joint analysis of allelic ratio and tumor sequence coverage (depth) at ∼1–3 million heterozygous germline SNP loci reflects the underlying somatic genotype of the tumor; (2) segmental regions of CNA and LOH span tens to thousands of contiguous SNP loci; (3) the observed sequencing signal is an aggregated measure of heterogeneous cellular populations, including normal and tumor subpopulations (Fig. 2A,D); and (4) sets of genetic aberrations observed at similar cellular prevalences possibly co-occurred in the same clone. We incorporated these assumptions into a two-chain factorial HMM (Fig. 2C; Methods; Supplemental Methods).

In order to evaluate and experimentally validate TITAN predictions, we used the genomes from a set of five synchronously resected pretreatment high-grade serous (HGS) ovarian cancer specimens (DG1136a,c,e,g,i) from the same patient. We obtained Illumina HiSeq 2500 WGS 100-bp paired-end data, sequenced at ∼30×, for each tumor sample and the patient’s matched normal DNA for a total of six data sets. There were ∼2.3 million high confidence heterozygous SNPs in the normal genome of DG1136. Across the five tumor samples, there were 2816 CNA/LOH events (Supplemental Table 1), with a range of event sizes (Supplemental Fig. 1) covering an average of ∼1.5M SNPs per sample. We used these data to generate two types of benchmarking data sets for which these original events formed the ground truth “positive set” in the quantitative evaluation. First, we simulated synthetic embedding of sampled CNA/LOH events into diploid chromosomes; and second, we systematically admixed the related but distinct CNA/LOH profiles from the tumor samples in known quantities to simulate mixed tumor populations. Finally, provision over the biological material allowed for experimental validation of TITAN predictions using FISH and single-cell sequencing.

### Simulated CNA spike-in experiment demonstrates accurate detection for varying event sizes

We profiled the CNA landscape of DG1136a using two complementary methods: HMMcopy (Ha et al. 2012) and Control-FREEC (Boeva et al. 2012). Both methods identified a large deletion (Chr16:46464744–90173515) and an amplification (Chr8:97045605–144155272) of interest (Supplemental Fig. 2; Supplemental Table 1A). From these events, we randomly sampled log ratios and allele counts for nonconsecutive sets of 10, 100, and 1000 positions. The sampled data were then inserted into diploid heterozygous chromosomes (Chr 1, 2, 9, and 18) at consecutive SNP positions to simulate segmental CNA and LOH events (Supplemental Methods). In total, there were four deletions and four amplifications for each event size. Median genomic sizes of these events were 6.9 kb, 82 kb, and 1.2 Mb. We also included one deletion and one amplification event spanning 10,000 SNPs (median 12.5 Mb) (Supplemental Table 2A). To vary cellular prevalences, we generated spike-in events sampled from two simulated tumor-normal admixtures at 80% and 60% of the original DG1136a data set, computationally admixed with its matched normal WGS data (Supplemental Methods; Supplemental Figs. 3–6).

TITAN was run, from a range of one to five clonal clusters, on the entire simulated sample (containing 78 events), including the chromosomes without spike-in events. The run with four clonal clusters was selected as optimal based on the *S*_*Dbw* validity index (Supplemental Methods; Halkidi et al. 2002). All 54 events of 100 SNPs or larger were detected (true positive rate [TPR] ≥ 0.9); however, only 11 of 24 events of 10 SNPs were recalled (Supplemental Table 2B). The global false positive rate (FPR) was 0.04, which was computed by considering all SNP positions in Chr 1, 2, 9, and 18 where no spike-in data was inserted.

The tumor cellular prevalence estimates for two of the TITAN clonal clusters were 0.52 and 0.36, which were within range of the expected values of 0.52 and 0.39 (Supplemental Methods). For deletions and amplifications, respectively, the cellular prevalence for 24 (89%) and 10 (37%) events with 100 SNPs or larger were correctly estimated (TPR ≥ 0.9) (Supplemental Figs. 3–6; Supplemental Table 2C). Despite the prevalence estimates of many amplifications not matching expected values, the events were still predicted to be subclonal but with a lower prevalence in some instances. On balance, the spike-in experiments demonstrate that TITAN is accurate at detecting (sub)clonal events of varying sizes, but illustrate a potential limitation in detection of very small (10 SNP) events and estimation of the true prevalence for amplifications (see Discussion).

### TITAN confers improved sensitivity for low-prevalence events in simulated tumor subpopulations

**Figure 3.**
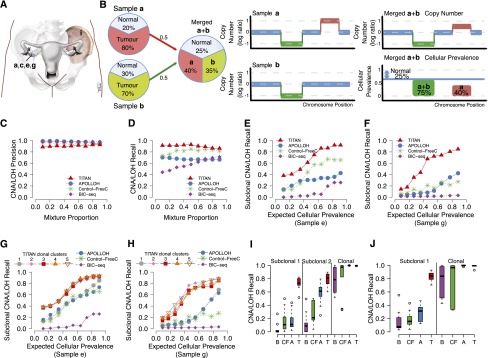
Performance of TITAN in serial and merging simulations using real intratumoral samples from a HGS ovarian carcinoma. (*A*) Patient DG1136 had biopsies synchronously resected from four sites in the primary tumor of the right ovary and one site from the left pelvic sidewall metastasis. (*B*) Illustration demonstrating the expected proportions in a simulation of two tumor subpopulations. The tumor content of Sample ***a*** (80%) and Sample ***b*** (70%) inform the sample cellular prevalence in the merged Sample ***a*** + ***b***. Events found in all samples of the mixture represent simulated clonal events. For example, the (green) deletion is present in 75% of the merged sample (or 100% of tumor cells) given that the normal proportion is 25%. Events present in a subset of samples in the mixture simulate subclonal events such as for the (red) gain unique to Sample ***a*** which is present in 40% of the merged sample or 53% of the tumor cells. (*C–F*) Performance of the serial mixture experiment between TITAN, APOLLOH (Ha et al. 2012) (which includes HMMcopy), Control-FREEC (Boeva et al. 2012), and BIC-seq (Xi et al. 2011). The mixture proportion includes 0.1:0.9, 0.2:0.8,…, 0.9:0.1 relative ratios of DG1136e:DG1136g. Precision (*C*) and recall (*D*) are shown for subclonal and clonal events averaged across gains, deletions, and LOH events. Recall performance for truth events found uniquely in Sample e (*E*) or Sample g (*F*) are shown. “Mixture Proportion” is defined as the ideal mixing fractions (e.g., 10%, 20%, etc.); expected tumor “cellular prevalence” is defined as the expected tumor contribution, at a given mixture proportion, from each individual sample making up the mixture. The expected tumor cellular prevalence shown was computed by adjusting the mixture proportion for tumor content of 67% and 56% for DG1136e and DG1136g, respectively. Ground truth events were identified in the individual samples of the mixture using APOLLOH/HMMcopy, and expected tumor cellular prevalence values are shown in Supplemental Table 3B. (*G,H*) Serial mixture performance for TITAN runs initialized with number of clusters ranging from one to five. Recall performance for events found uniquely in DG1136e (*G*) or DG1136g (*H*) represent events that are subclonal within the simulated mixture. Average recall across deletions, gains, and LOH events are shown. The one-cluster run represents the scenario in which only one tumor population exists. (*I,J*) Comparison of recall performance distributions across 10 paired (*I*) and 10 triplet (*J*) merging simulations for TITAN (T), APOLLOH/HMMcopy (A), and Control-FREEC (CF). Performance is shown for simulated subclonal events, which were present uniquely in exactly one (Subclonal 1) and exactly two (Subclonal 2) samples making up the mixture; and in contrast, clonally dominant events were present in all samples of the mixture (Clonal).

To assess the performance of TITAN using benchmarking data sets that are more representative of clonal mixtures, we designed systematic experiments that simulated genomes with multiple tumor subpopulations at known proportions. In silico, we mixed the WGS data from DG1136a,c,e,g,i (Fig. 3A; Supplemental Table 3A). Within each mixture, we defined *clonal* events as CNA/LOH events present in all individual samples and *subclonal* events as present in only a subset of the samples (Fig. 3B; Supplemental Table 3B–D). The proportion of tumor contribution from each individual sample (Supplemental Table 3A) in the mixture was used to compute the expected cellular prevalence (Supplemental Methods).

We combined DG1136e (67% tumor cellularity) and DG1136g (56% tumor cellularity), at mixture proportion increments of 10% (Methods), resulting in nine (∼30×) mixtures with two simulated tumor populations at 0.07/0.50, 0.13/0.45, 0.20/0.39, 0.27/0.33, 0.33/0.28, 0.40/0.22, 0.47/0.17, 0.53/0.11, and 0.60/0.06 relative ratios (Supplemental Table 3B). Figure 3B illustrates a mixture scenario, which identifies true (sub)clonal events and their expected cellular prevalence. We compared accuracy of detection of CNA and LOH events using TITAN (run once each for a fixed number of clusters ranging from one to five), APOLLOH (A) (Ha et al. 2012), Control-FREEC (CF) (Boeva et al. 2012), and BIC-seq (B) (Xi et al. 2011). After selecting the optimal number of clusters using the *S*_*Dbw* validity index (Methods), TITAN’s median overall F-measure over the nine mixtures for predicted clonally dominant and subclonal events was 0.90. This was similar to APOLLOH (0.91) and Control-FREEC (0.88), but higher than BIC-seq (0.73) (Supplemental Fig. 7A; Supplemental Table 4A). Although the precision for all approaches performed comparably (Fig. 3C), TITAN had higher sensitivity—median 0.91 compared to 0.85 (A), 0.83 (CF), and 0.58 (B), respectively (Fig. 3D).

TITAN’s sensitivity gains could be primarily attributed to improved sensitivity to subclonal events (Fig. 3E,F). Accordingly, we observed improved performance for runs with two or more clusters, but not when run with one cluster (Fig. 3G,H). Over the range of two to five clusters, recall was similar for subclonal events, suggesting TITAN is relatively stable in its predictions when accounting for more than one tumor subpopulation. Despite this stability, we explored the utility of unbiased model selection to choose the optimal number of clusters. We note that three clusters fit the scenario in which events may be clonally dominant (present in both samples) or subclonal, having one of two possible unique cellular prevalences contributing from the individual samples. Using the *S*_*Dbw* validity index, three clusters were appropriately selected as the optimal number for the majority of the mixtures (Supplemental Table 3B). The subclonal predictions using the optimal cluster runs consistently outperformed the other methods (Fig. 3E,F). In addition, performance gains were maintained across ranges of event lengths of 10–100 kb, 100 kb–1 Mb, 1–10 Mb, and >10 Mb (Supplemental Fig. 8).

Next, we used the DG1136 data sets to generate 10 pairwise (61–69× coverage) and 10 triplet (95–103× coverage) merged combinations of the five intratumor samples, mixed at approximately equal proportions (Supplemental Table 3C,D). In the triplet-merged samples (Supplemental Fig. 9; Supplemental Table 4B), TITAN performed comparably for all (clonal and subclonal) amplifications (0.85 median F-measure) relative to APOLLOH (0.85), Control-FREEC (0.85), and BIC-seq (0.60). For deletions and LOH events, TITAN showed statistically significant improvement over the other algorithms (0.91 compared to 0.87 [A], 0.83 [CF], and 0.60 [B]; two-sample Wilcoxon rank-sum test *P* < 0.001) and LOH events (0.96 compared to 0.94 [A] and 0.85 [CF]; *P* < 0.001). Similar performance was observed in the pairwise-merged samples with comparable F-measure for all amplifications (median 0.87 compared to 0.87 [A], 0.85 [CF], and 0.70 [B]) and statistically significant improvement in F-measure for deletions (0.95 compared to 0.92 [A], 0.87 [CF], and 0.69 [B]; *P* < 0.005) and LOH events (0.98 compared to 0.96 [A] and 0.89 [CF]) (Supplemental Fig. 10; Supplemental Table 4C).

As shown for the serial mixture simulation, TITAN was more sensitive for subclonal events than the other methods in both pairwise and triplet merging simulations. Events unique to only one sample (subclonal in the mixture) were predicted with statistically higher recall by TITAN (Fig. 3I,J) (two-sample Wilcoxon rank-sum tests, *P* < 0.001). TITAN was also more sensitive to subclonal deletion and LOH events present in exactly two samples in the triplet-merged mixture (*P* < 0.001), whereas amplifications were comparable (Fig. 3I). All methods accurately predicted clonally dominant events for each merged simulation. Therefore, while maintaining accuracy of clonal events, TITAN showed clear advantages in detection of the engineered subclonal events. We attribute this increase in sensitivity directly to the consideration of heterogeneous cell populations in the model.

### Accurate estimation of cellular prevalence and normal contamination

**Figure 4.**
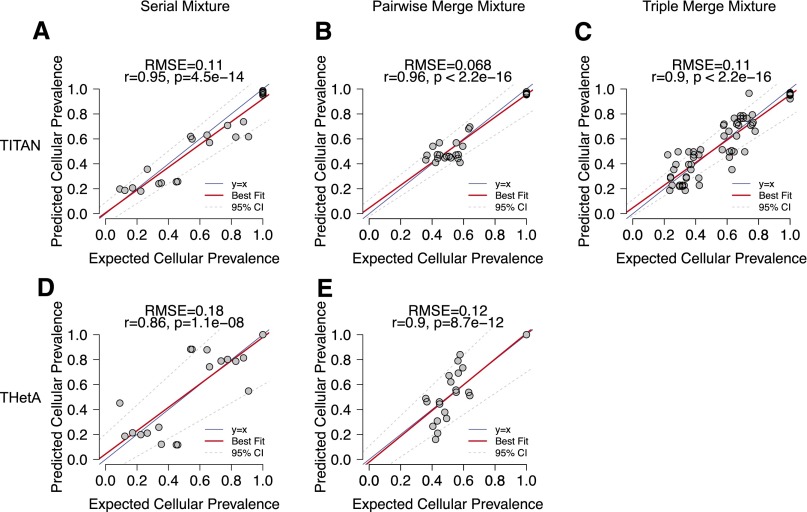
Performance of TITAN tumor cellular prevalence estimates for serial (30×) and pairwise (60×)/triplet (90×) merging simulations of intratumor samples from a HGS ovarian carcinoma. Pearson correlation coefficients (*r*) and root mean squared error (RMSE) were computed for TITAN (*A–C*) and THetA (Oesper et al. 2013) (*D*,*E*). Correlation and RMSE were computed by comparing the cellular prevalences of the predicted clusters with the prevalence of the expected clusters across the mixture samples. Each data point represents an expected clonal cluster with a unique tumor cellular prevalence. Ground truth and expected tumor cellular prevalence values were computed from the tumor contribution from each individual sample making up the simulated mixture (Supplemental Table 3B–D).

To evaluate the accuracy of cellular prevalence predictions (one of the key parameters estimated by TITAN), we compared the expected ground truth values for each simulated mixture. For each pairwise mixture, three clonal clusters were expected (one ancestral and two sample-specific), and for triplet mixtures, up to seven clonal clusters (all possible combinations of three samples) were expected. The expected cellular prevalence was computed using the tumor contribution from each individual sample making up the simulated mixture (Supplemental Methods; Supplemental Table 3B–D). Cellular prevalence estimates predicted by TITAN showed high and statistically significant positive correlation (Pearson’s *r* ≥ 0.9, *P* < 0.001, root mean squared error [RMSE] ≤ 0.11) with the expected tumor cellular prevalence across all samples in the serial (Fig. 4A; Supplemental Table 3B) and merging simulations (Fig. 4B,C; Supplemental Table 3C,D), demonstrating that the model was able to reproduce the engineered clonal structure.

Next, we compared cellular prevalence estimates between TITAN and THetA (Oesper et al. 2013). THetA’s estimates also showed statistically significant correlation with expected values (Pearson’s *r* > 0.86, *P* < 0.001) (Fig. 4D,E); however, the RMSE was lower for TITAN (0.11) compared to THetA (0.18) for the serial mixtures and similarly for the pairwise mixtures (0.07 compared to 0.12). Due to time complexity limitations, we were only able to run THetA for up to two tumor populations, therefore comparison on the triplet mixtures could not be performed. THetA has superpolynomial time and memory complexity when two or more tumor subpopulations are considered. Moreover, complexity increases with the number of input regions; thus, fewer than 15 segments were required for reasonable runtimes, resulting in lower resolution when inferring subclonal events. Finally, we also used an orthogonal approach (Control-FREEC) to estimate tumor content used to compute the expected cellular prevalence and observed similar correlations and RMSE results for TITAN and THetA (Supplemental Fig. 11; Supplemental Table 5).

Global normal contamination impacts the ability of the model to reconcile the presence of subclonality. We assessed the ability of the model to correctly estimate the global normal contamination in the model. TITAN estimates showed significantly positive correlation for the serial mixtures (Pearson’s *r* = 0.96, *P* < 0.0001, RMSE = 0.023), pairwise mixtures (*r* = 0.86, *P* = 0.0014, RMSE = 0.047), and the triplet mixtures (*r* = 0.74, *P* = 0.014, RMSE = 0.048) relative to the expected normal proportion (Supplemental Fig. 12A–C; Supplemental Table 3B–D). TITAN’s estimates were considerably more accurate than THetA for the serial (*r* = 0.93, *P* < 0.0002, RMSE = 0.23) and pairwise mixtures (*r* = 0.51, *P* = 0.14, RMSE = 0.3) (Supplemental Fig. 12D,E). Therefore, in addition to increased sensitivity for detecting subclonal events, TITAN showed accurate inference of cellular prevalence and normal proportion, indicating the model formulation more closely models the mixture of cells generating the data. This not only contributes to increased accuracy in detection, but also adds an interpretive layer to estimating the composition of both tumor and normal cells being sequenced.

### Characterization of the subclonal copy number alteration landscape in triple negative breast cancers

Having established quantitative accuracy with benchmarking data sets, we next analyzed a set of 23 triple negative breast cancers (TNBC) with paired tumor-normal whole-genome sequencing (Ha et al. 2012; Shah et al. 2012). We applied TITAN to predict regions of (sub)clonal CNA and LOH in the TNBC genomes (Supplemental Table 6) and profile the patterns of cell population structure inferred from the genome architecture. Six cases were clonally homogeneous (i.e., one clonal cluster) with cellular prevalence between 0.91 and 0.97, whereas the remaining 17 cases were more heterogeneous (between two and six clonal clusters) and cellular prevalence estimates ranging from 0.17 to 0.98 (Supplemental Table 7A). In the 17 heterogeneous cases, the proportion of the genome altered by subclonal events ranged from 0.16 and 0.73, with 12 cases having a higher proportion of subclonal alterations than clonal events (Supplemental Table 7B). This emphasizes the importance of considering mixed populations, which if neglected will lead to vastly different interpretations of the data and preclude inference of evolutionary patterns.

For four of the TNBC cases, whole-exome capture data was also available. We applied TITAN to this exome sequencing data to demonstrate that the method can also be used for data for targeted genomic regions. Across the four samples, there were 79,097 (∼19,700 per sample) overlapping SNP positions between the exome and WGS data, of which 55,846 (71%) were concordant for predicted copy number between the TITAN results for the two data types (Supplemental Fig. 13; Supplemental Table 8). However, in the WGS data, TITAN appears to resolve the signal into a potentially higher number of clonal clusters, possibly due to 100× more SNP loci (Supplemental Table 8).

For 22 of the TNBC cohort, we also analyzed the transcriptomes sequenced via RNA-seq (Ha et al. 2012; Shah et al. 2012) to assess whether the clonality of LOH predictions influences allele-specific expression. Using the transcriptome allelic ratio (TAR; proportion of reference read counts) as an orthogonal measure, we compared TITAN cellular prevalence estimates to expected allelic imbalance in expression. We considered only RNA-seq positions within deletion LOH segments and estimated the expected baseline TAR as a function of the cellular prevalence 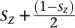.

The first term corresponds to the tumor cells having only one copy or allele, and the second term represents all other cells without the LOH event (i.e., diploid and heterozygous). Across the 22 TNBC cases and each clonal cluster, the TAR was significantly correlated with the predicted cellular prevalence (Pearson’s *r* = 0.71, *P* = 1.5 × 10^−10^) (Supplemental Fig. 14A). TAR values were observed to be more imbalanced than could be explained by deletion LOH alone. This could be attributed to epigenetic silencing of one or both alleles in cells without LOH. When higher copy number events are considered, such as amplified LOH, this can also result in more imbalance than expected due to stronger representation of the homozygous allele if expression is positively correlated with copy number (Supplemental Fig. 14B). These results indicate that a substantial proportion of monoallelic expression is associated with coincident subclonal LOH prediction, providing evidence that despite the presence in only a minor cell population, these events are impacting the transcriptional program in these tumors.

### FISH assays validate the presence of subclonal copy number changes

**Figure 5.**
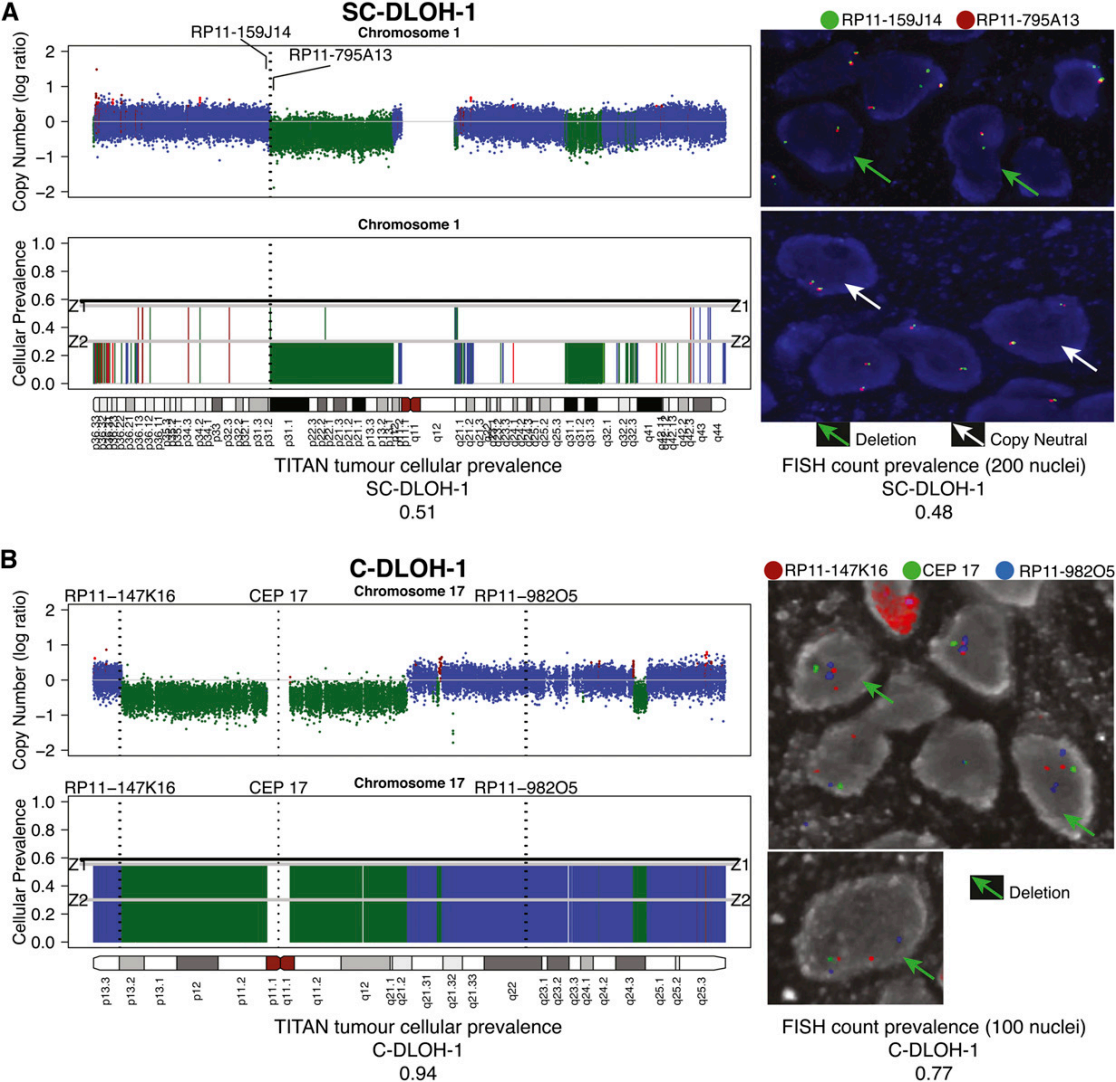
Fluorescence in situ hybridization (FISH) validation of TITAN predictions for Chromosomes 1 and 17 in DG1136g. (*A*) Subclonal hemizygous deletion, SC-DLOH-1, in Chromosome 1 was validated using BAC probe RP11-795A13 (orange, Chr1:69851036–70025173). Control probe for copy neutral regions was RP11-159J14 (green, Chr1:69454844–69606688). FISH imaging shows tumor cells with a deletion (green arrow) and diploid (white arrow) at this region. (*B*) Clonal deletion, C-DLOH-1, in Chromosome 17 was validated using the centromeric probe, CEP 17. The BAC probes RP11-147K16 (orange, Chr17:3294803–3452243) and RP11-982O5 (blue, Chr17:55475584–55662513) were used as controls. The majority of cells were observed to harbor the deletion. FISH count prevalence was computed as the proportion of nuclei with event:control count ratio that is <1 (deletion) or >1 (gain) (Supplemental Table 9H). FISH imaging is shown at 63× magnification. Copy number predictions are shown using log ratios (normalized tumor depth/normal depth). Copy neutral (blue), hemizygous deletion (green), and copy gain (red) predictions are shown. Cellular prevalence estimates for clonal cluster 1 (Z1) and cluster 2 (Z2) predicted by TITAN are shown; tumor cellularity is indicated by the black horizontal line.

We performed fluorescence in situ hybridization (FISH) assays in two primary tumor HGS ovarian cancer samples, DG1136c and DG1136g, using cryosections from the same tissue blocks used for WGS (Supplemental Methods). We targeted seven TITAN CNA predictions (Supplemental Table 9A): one high-prevalence deletion (C-DLOH-1) (Fig. 5), four low-prevalence deletions (SC-DLOH-1, 3, 4, and 5) (Supplemental Figs. 15, 16), and one low-prevalence gain (SC-GAIN-1) (Supplemental Fig. 15) in DG1136g. An additional low-prevalence gain (SC-GAIN-2) was assayed in sample DG1136c. Notably, all six low-prevalence events were missed (detected as neutral regions) by at least one of the three other comparison methods: HMMcopy, Control-FREEC, and THetA (Supplemental Table 10). Each event was scored by counting 100 or more nuclei to obtain a quantitative measure of the prevalence (Supplemental Table 9B–G).

The presence of all seven events was confirmed by FISH (Supplemental Table 9H). Quantitative estimates of prevalence by FISH were broadly consistent with TITAN predictions (Supplemental Table 10). The highest prevalence event, C-DLOH-1 (Chromosome 17 centromere), which represented a clonal deletion at 0.94 TITAN cellular prevalence (and predicted by all other methods), was observed in 0.77 of cells (Fig. 5; Supplemental Table 9H). SC-GAIN-2 (11q13.1), which had TITAN cellular prevalence of 0.62, was present in 0.61 of the nuclei scored by FISH in DG1136c. The remaining five events had predicted a cellular prevalence of 0.51 in DG1136g. Two events, SC-DLOH-1 (1p31.1) and SC-DLOH-3 (2p16.1), were both observed at a FISH prevalence of 0.48. These events were not predicted by Control-FREEC. SC-GAIN-1 (2p23.2), which was not detected by THetA, had an observed FISH prevalence of 0.35. Neither SC-DLOH-4 (7q35) nor SC-DLOH-5 (21q21.1), which were observed at FISH prevalences of 0.26 and 0.36, were predicted by HMMcopy, Control-FREEC, or THetA (Supplemental Table 10). Thus, experimental revalidation using cytogenetic techniques on cryopreserved patient material confirmed low- and high-prevalence gains and deletions predicted by TITAN, emphasizing the enhanced sensitivity conferred by modeling the presence of multiple populations.

### Validation of TITAN predictions using single-cell sequencing confirm the presence of multiple tumor populations

We further validated the CNA predictions from DG1136g using single-cell sequencing of targeted positions. The nuclei were isolated and sorted from disaggregated frozen tissue blocks and sequenced using multiplex PCR reactions and Fluidigm access array technology (Supplemental Methods). Two sets of events, Set1 and Set2 (Supplemental Tables 11A, 12A), each included one high-prevalence clonal LOH event, two subclonal deletions, and two heterozygous diploid regions (Supplemental Fig. 17). For each set, 42 single cells were sorted, followed by library construction and sequencing; statistical analysis was then carried out independently for the two sets (Supplemental Methods).

**Figure 6.**
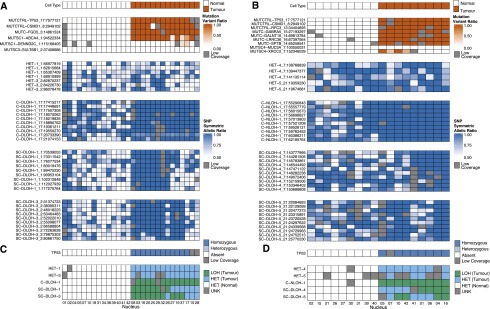
Single-cell validation of subclonal deletions in DG1136g using deep DNA sequencing of individual nuclei. (*A,B*) The 28 nuclei for Set1 and 18 nuclei for Set2 were designated as tumor and normal cell type using the status of mutations. The mutant allele ratio (*variant reads*/*depth*) for mutations and symmetric allele ratio (*max*(*reference reads*, *variant reads*)/*depth*) for SNP positions are shown for Set1 (*A*) and Set2 (*B*) events. Low coverage positions are shaded in gray. (*C,D*) The LOH status for each event for Set1 (*C*) and Set2 (*D*) were determined using the binomial test for dropout and Wilcoxon rank sum test for allelic ratios. *TP53* mutation status is shown. The LOH status for each heterozygous (HET) and LOH (C-DLOH, SC-DLOH) event is shown. “Tumor” nuclei having the LOH event (green) or not having the event (blue) are shown to illustrate the original three-component mixture model (Fig. 2A). Normal nuclei are designated “Normal” (white). Unknown events (gray) were inconclusive for HET or LOH status. See Supplemental Methods for details.

This experiment focused on LOH events because confirmation of homozygosity (the absence of one allele) in single-cell sequencing is generally unambiguous. For statistical robustness, we interrogated multiple SNPs within each prediction of LOH (10–11 SNPs) and heterozygous (2–3 SNPs) negative control regions. We also selected previously validated somatic point mutations (SNVs), including a homozygous SNV in *TP53*, from this tumor. Because it is widely accepted that *TP53* mutation is a tumor-initiating event in HGS ovarian cancer (Ahmed et al. 2010; The Cancer Genome Atlas Research Network 2011; Bashashati et al. 2013), this mutation was expected to be present in all tumor cells. *TP53*, along with the other SNVs, were used as markers to distinguish tumor and contaminating normal nuclei in this experiment (Supplemental Methods). This resulted in 14 tumor and 14 normal nuclei for Set1 (Fig. 6A; Supplemental Table 11B), and nine tumor and nine normal nuclei for Set2 (Fig. 6B; Supplemental Table 12B). The remaining nuclei contained insufficient read coverage for analysis.

For predicted clonal LOH events, we expected to observe homozygous signals for SNPs in all tumor nuclei. In contrast, for subclonal events, we expected homozygous SNPs to be present in only a subfraction of tumor nuclei. We used two statistical tests (Methods) to determine if an LOH event in a nucleus was present across the set of positions in the event. This involved controlling for expected allele dropout frequency (from unequal amplification of alleles) inferred from the normal nuclei at the predicted heterozygous loci. Over the set of positions in an event, we classified each nucleus as heterozygous or homozygous (or unknown if statistically inconclusive). As expected, for each of the normal nuclei in both Set1 and Set2, all LOH events were classified as heterozygous, independently confirming the initial grouping of cell types using mutations. In addition, the four negative control heterozygous events HET1, HET3 (Fig. 6A,C), HET4, and HET5 (Fig. 6B,D) were each classified as heterozygous in all tumor nuclei for which sufficient coverage was obtained. In contrast, for the predicted clonal LOH events C-DLOH-1 (Fig. 6A,C) and C-NLOH-1 (Fig. 6B,D), all tumor nuclei were classified as homozygous, confirming that the LOH predictions were clonally dominant. For each of the predicted subclonal deletion events (SC-DLOH-1, 3, 4, and 5), the tumor nuclei were divided into two groups with homozygous and heterozygous status, respectively. The proportions of tumor nuclei with homozygous status in these events were 0.54 (7/13 for SC-DLOH-1), 0.71 (10/14 for SC-DLOH-3), 0.50 (4/8 for SC-DLOH-4), and 0.50 (4/8 for SC-DLOH-5), which were generally consistent with the TITAN cellular prevalence estimate of 0.51 (Supplemental Table 3E). Therefore, in two independently executed single-cell sequencing experiments, we were able to relate our predictions back to the key modeling assumptions of TITAN and confirm the presence of the three cell types (Fig. 2A): (1) a population of normal cells; (2) a population of tumor cells harboring the CNA/LOH event; and (3) a population of cells without the CNA/LOH event.

## Discussion

TITAN is a novel algorithm that jointly analyzes both the tumor read depth and digital allele read counts for segmentation of subclonal CNA and LOH in whole-genome sequencing of tumors. The advantages of TITAN are threefold. First, the proper deconvolution of signals in the sequencing reads using the proposed sampling model allows for improved performance for predicting CNA and LOH (Fig. 3C,D). Second, the algorithm is more sensitive to subclonal events, which generally have more diluted signals, demonstrated by the serial and merging mixture experiments from a HGS ovarian cancer data set (Fig. 3E–J). Third, estimation of tumor cellular prevalence and normal proportion is a powerful feature that enables inference of evolutionary clonal dynamics of the tumor’s genome at CNA and LOH scales (Fig. 4; Supplemental Fig. 12). Importantly, using deep sequencing of single nuclei, we were able to successfully confirm the presence of six (sub)clonal LOH and two subclonal copy number gain events. FISH experiments confirmed that TITAN was able to detect low-prevalence events that were not predicted by three other methods. In summary, results from experiments over a broad range of synthetic data, patient tumor data, and experimental validation have established that, through appropriate statistical modeling, the composition of cell populations in source tumor samples can be accurately identified by inference of CNA and LOH events in WGS data, and consideration of mixed population leads to enhanced accuracy.

We demonstrated single-cell analysis as a viable experimental validation for unambiguously confirming the presence of TITAN-predicted (sub)clonal LOH events at the resolution of individual cells. The quantification of cellular prevalence was still challenging due to the small numbers of nuclei, and inference of patterns of clonal evolution was limited by the analysis of only three LOH events in each cell. Scaling up the numbers of tumor nuclei and LOH events will enable the statistical analysis of mutual exclusion and co-occurrence of events that are unattainable from single bulk tumor biopsies and construction of clonal evolutionary phylogenies (Potter et al. 2013). More conventional FISH experiments also corroborated TITAN predictions, including low-prevalence deletions and gains. Interestingly, for two events, prevalences determined by FISH were lower (0.26 and 0.36) than TITAN cellular prevalence estimates. Although TITAN may have over-clustered them into a higher prevalence clonal cluster, the presence of these predicted CNAs was still confirmed by FISH. Moreover, the lack of prediction of these events by any of the other competing methods illustrates TITAN’s enhanced sensitivity. These results also suggest that ∼0.30 prevalence may fall below the model’s ability to accurately resolve the clustering for samples sequenced at ∼30×. Thus, higher sequence coverage may be required for greater accuracy in cellular prevalence estimates.

TITAN has several limitations due to its specific modeling assumptions. TITAN does not model more than one aberrated genotype at the same locus, but instead assumes that clones harboring the subclonal event coexist with tumor population(s) that have a normal (diploid heterozygous) genotype. An example of this limitation is shown in Figure 1, in which the event at Chr 2q23.3–q24.1 is likely an aggregated signal from a tumor population with a hemizygous deletion and another with a copy neutral LOH (subsequent duplication). In particular, it is difficult to distinguish among coexisting clones that harbor amplifications of variable copies. In order to model multiple tumor genotypes, the mixture representation model (Fig. 2A) will need to be reformulated; yet given only tumor read depth and allele counts, the joint analysis of these signals may yield multiple solutions.

The primary aim of TITAN is the improved sensitivity for detecting subclonal CNA/LOH events, and we have demonstrated this performance in benchmarking mixtures up to ∼90× sequencing coverage (Figs. 3I, 4C); however, this is limited to resolving events in major clones at detectable prevalences. The full enumeration of clonal cell populations, including minor clones, is a limitation of TITAN and remains a difficult problem in the analysis of single tumor biopsies. This was demonstrated with FISH, which revealed the presence of rare, isolated nuclei harboring genotypes not detectable by TITAN. Solutions to address these limitations will require integration of additional data, such as somatic SNVs (Carter et al. 2012; Fischer et al. 2014; Roth et al. 2014), genomic rearrangement breakpoints, increased coverage of sequencing (Nik-Zainal et al. 2012), multipatient biopsies (Bashashati et al. 2013; Gerlinger et al. 2014), and ultimately, single-cell analysis (Navin et al. 2011; Potter et al. 2013).

TITAN estimates parameters using the EM algorithm; however, this approach may return locally optimal solutions influenced by the initializations prior to inference. Informed initializations of the key parameters, normal proportion and tumor ploidy, using orthogonal sources provided by histopathology may improve EM-converged parameter values and overall predictions. Also, we determined the optimal number of clonal clusters that best represented the expected number of clonal groups in the sample by using a modified internal evaluation measure *S*_*Dbw*. A more robust solution would be to integrate the model selection directly into the framework, such as representing the clonal cluster groupings using a phylogenetic tree to relate inferred clones into their ancestral lineages.

Our results indicate that neglecting to model WGS data as a composite of different tumor populations with diverse somatic genomes results in an incomplete representation of a tumor’s CNA and LOH landscapes. This is exemplified by the analysis of the TNBC genomes, which suggested that the somatic copy number architecture in tumors might have evolved substantially. It was recently shown that the inference of subclonal mutations impacts the outcome of chronic lymphocytic leukemia under treatment-based selective pressures (Landau et al. 2013). Whether similar outcome correlations are detectable when CNA and LOH are the aberrations measured for evolutionary patterns remains an open question. We anticipate that the TITAN framework will provide a robust analytical route for studying the degree to which clonal evolution, driven from the CNA and LOH perspective, influences treatment sensitivity.

A downstream clinical application of TITAN will be the identification of tumors with DNA repair defects that would make them susceptible to genotoxic drugs. Compromised homologous recombination (HR) due to loss of *BRCA1/2* is a dominant molecular feature of HGS ovarian cancer (The Cancer Genome Atlas Research Network 2011) and other epithelial malignancies (Yang et al. 2013). HR defects lead to accrual of genomic structural changes through unrepaired double-strand genomic breaks (Lord and Ashworth 2012). HR-deficient cells carry the genomic footprints of progressive accumulation of structural genomic aberrations, including regions of CNA and LOH. Tumors exhibiting subclonal CNA and LOH events are likely to have acquired HR defects and would be good candidates for treatment with platinum-based drugs or PARP inhibitors (Mukhopadhyay et al. 2012; Wang et al. 2012). Large-scale whole-genome sequencing studies of epithelial cancers coupled with reliable homologous recombination deficiency assays will be an appropriate way to test this hypothesis.

In conclusion, the TITAN statistical framework represents a significant advance in the field of copy number and LOH analysis for tumor genome sequencing data. The implications for clonal evolution of point mutations in clinical trajectories are well documented; we suggest that TITAN will enable the execution of complementary studies to investigate the role of genome architecture in driving the evolutionary selection of clonal cell populations.

## Methods

### The TITAN statistical model

To model tumors containing multiple tumor subpopulations, we assumed the observed measurements were generated from a composite of three types of cell populations (Yau et al. 2010) with relative proportions as follows: *n*: the proportion of nonmalignant cells; (1 − *n*)*s*_*z*_: the proportion of tumor cells with normal genotype; and (1 − *n*)(1 − *s*_*z*_): the *sample cellular prevalence* or the proportion of tumor cells harboring the CNA or LOH event of interest (Fig. 2A). *s*_*z*_ is the proportion of tumor cells that is diploid heterozygous (and therefore normal) at the locus. Thus, (1 − *s*_*z*_) is the *tumor cellular prevalence* or the proportion of the tumor population containing the event. We assume multiple somatic events share similar cellular prevalence and thus can be assigned to one of a finite number of clonal clusters, *z* ∈ *Z*. This allows for sufficient data points to robustly infer the model parameters by borrowing statistical strength. The simultaneous inference and clustering of each data point to *z* ∈ *Z* is the primary distinguishing feature over related work (Van Loo et al. 2010; Yau et al. 2010; Carter et al. 2012; Oesper et al. 2013; Yau 2013).

The inputs to the model are quantities readily extracted from WGS sequence data (Fig. 2B). The analysis requires the genome-wide set of *T* germline heterozygous SNP positions derived from the normal genome, which generally ranges from 1 to 3 million per patient. At each SNP, copy number data from the tumor genome is represented by the log ratio between the tumor and normal read depths *l*_1:*T*_. We assume *l*_1:*T*_ is Gaussian distributed: 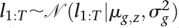. We assume the reference allelic read counts from the tumor *a*_1:*T*_ are binomial distributed *a*_1:*T*_ ∼ *Bin*(*a*_1:*T*_|*N*_1:*T*_, *ω*_*g*,*z*_), where *N*_1:*T*_ represents the sequencing depth at each position. The cluster-specific parameters *μ*_*g*,*z*_ and *ω*_*g*,*z*_ are functions of *s*_*z*_ (Fig. 2D), and therefore represent the signals from the three types of cell populations. This formulation enables TITAN to be more sensitive to events with lower cellular prevalences.

Segmental CNA and LOH events span many contiguous SNP positions, thereby inducing spatial correlation along the chromosome. To capitalize on expected shared signals from adjacent positions, TITAN was implemented as a two-factor hidden Markov model (HMM) in which the hidden genotypes *G*_1:*T*_ and the hidden clonal cluster memberships *Z*_1:*T*_ comprise the two chains (Fig. 2C). The state space is dynamically expanded as a function of clonal cluster membership, resulting in |*G*| × |*Z*| number of state tuples (*g* ∈ *G*, *z* ∈ *Z*) (Supplemental Table 14). The HMM is fit to the data using expectation maximization (EM) as described in the Supplemental Methods.

The final output of TITAN is a list of segment boundaries that represent CNA and LOH events with accompanying estimates of the genotype, the cellular prevalence (1 − *s*_*z*_), and clonal population cluster membership for each event. In addition, estimation of global parameters *n*, the normal proportion, and *φ*, the estimated ploidy, are output. The parameters of the probabilistic graphical model (Fig. 2C) are defined in Supplemental Table 13, and full mathematical details are described in Supplemental Methods.

### Analysis workflow

The analysis workflow of TITAN for tumor whole-genome sequencing data is shown in Figure 2B. First, germline heterozygous SNP positions 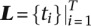 are identified from the normal genome using SAMtools *mpileup* (Li et al. 2009). The analysis focuses on ∼1–3 million loci genome-wide per patient and allows for identification of somatic allelic imbalance events (Ha et al. 2012). From the tumor genome data, the read counts mapping to the reference base (*A* allele) and total depth at all positions in ***L*** are extracted and represented as *a*_1:*T*_ and *N*_1:*T*_, respectively.

The tumor copy number is normalized for GC content and mappability biases using only the normalization component of HMMcopy (http://bioconductor.org/packages/2.11/bioc/html/HMMcopy.html). Briefly, the genome is divided into bins of 1 kb, and read count is represented as the number of reads overlapping each bin. Loess curve fitting and correction was performed on tumor and normal samples, separately. The corrected read counts for the overlapping 1-kb bin at each position of interest *t* ∈ ***L***, 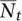, and 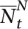 is used to compute the log ratio, 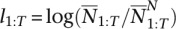.

TITAN jointly analyzes the data *l*_1:*T*_, *a*_1:*T*_, *N*_1:*T*_ to segment the data into regions of CNA/LOH and estimate normal contamination, tumor ploidy, and cellular prevalences for *Z* number of clonal clusters. For a range of *i* = 1 to 5, TITAN is run once for the set of clonal cluster states *Z*_*i*_ := {1, …, *i*}, where |*Z*_*i*_| = *i* is the number of clonal clusters. The optimal number of clusters *i* is then chosen using the minimum *S*_*Dbw* validity index (Supplemental Methods).

### In silico mixture experiments simulating multiple tumor subpopulations

Five intrapatient samples from patient DG1136 were used to simulate multiple cellular populations by mixing combinations of samples at known proportions. For the predefined serial mixture experiment, nine whole-genome mixtures at ∼30× coverage were generated by sampling reads from DG1136e and DG1136g at mixing proportions of 10% increments (0.1e/0.9g, 0.2e/0.8g,…, 0.8e/0.2g, 0.9e/0.1g). The expected relative tumor content contributions from the two samples were computed for each mixture based on tumor cellularity of 67% and 56%, respectively, as consensus estimates by APOLLOH and the pathological review (Supplemental Table 3B). HMMcopy and APOLLOH (Ha et al. 2012) results from the individual samples were used as ground truth CNA and LOH events, respectively, with default parameters (http://compbio.bccrc.ca/software/apolloh). For the merging of two or three samples at approximately equal proportions, five intratumor samples were merged together to generate 10 pairs at ∼60× coverage (Supplemental Table 3C) and 10 triplets at ∼90× coverage (Supplemental Table 3D) for each combination. This was done using SAMtools (Li et al. 2009) merge command.

Precision, recall, and F-measure were computed based on copy number status at heterozygous germline SNP positions from the individual samples (prior to mixing) predicted by APOLLOH/HMMcopy. Performance was calculated for deletions, gains, and LOH independently and averaged together for overall assessment shown in Figure 3C–J. The number of SNPs for ground truth deletion, amplification, and LOH events used to calculate performance metrics are given in Supplemental Tables 1, 3, and 4. See Supplemental Methods for more details.

### Statistical tests for single-cell sequencing experiments

Two statistical tests were used to determine if an event in a nucleus was statistically significant for LOH. First, we addressed allelic dropout, which is the preferential amplification of one allele at a heterozygous locus, leading to a homozygous signal that may be mistaken for LOH. Using the expected allelic dropout rate (*DOR*) of 0.28 (Set1) and 0.48 (Set2) determined from the normal nuclei (Supplemental Tables 11C, 12C), we applied a one-tailed binomial test in which the null hypothesis asserts that the proportion of homozygous positions is not greater than the expected *DOR*. The second test examined whether the allelic ratio distribution (Fig. 6A,B) across the positions for an event showed a statistically significant difference compared to the expected heterozygous allelic ratio (*HAR*) as determined from the normal nuclei. Finally, the maximum of the (Benjamini and Hochberg [FDR] adjusted) *P*-values between the two tests was used to determine if an event was statistically significant (FDR < 0.05) for LOH status, or heterozygous (HET) otherwise; events were designated as unknown or ambiguous for nonsignificant FDR and absence of a heterozygous position (Supplemental Tables 11E,F, 12E,F; Benjamini and Hochberg 1995).

Additional methods on mathematical details of the TITAN model, the inference algorithm, and software as well as experimental protocols for generating the validation data are provided in the Supplemental Methods.

## Data access

The ovarian cancer genome sequence data, including the single-cell data, have been submitted to the European Genome-phenome Archive (EGA; https://www.ebi.ac.uk/ega/) under accession number EGAS00001000547. TITAN is available at http://compbio.bccrc.ca/software/titan/ and can be downloaded from Bioconductor under the R package, TitanCNA.
